# Heat or Insulation: Behavioral Titration of Mouse Preference for Warmth or Access to a Nest

**DOI:** 10.1371/journal.pone.0032799

**Published:** 2012-03-30

**Authors:** Brianna N. Gaskill, Christopher J. Gordon, Edmond A. Pajor, Jeffrey R. Lucas, Jerry K. Davis, Joseph P. Garner

**Affiliations:** 1 Department of Animal Science, Purdue University, West Lafayette, Indiana, United States of America; 2 Charles River Laboratories, Wilmington, Massachusetts, United States of America; 3 Environmental Protection Agency, Research Triangle Park, North Carolina, United States of America; 4 Department of Production Animal Health, University of Calgary, Calgary, Alberta, Canada; 5 Department of Biological Sciences, Purdue University, West Lafayette, Indiana, United States of America; 6 Department of Comparative Pathobiology, Purdue University, West Lafayette, Indiana, United States of America; 7 Department of Comparative Medicine and the Department of Psychiatry and Behavioral Sciences, Stanford University, Stanford, California, United States of America; Université de Bordeaux and Centre National de la Recherche Scientifique, France

## Abstract

In laboratories, mice are housed at 20–24°C, which is below their lower critical temperature (≈30°C). This increased thermal stress has the potential to alter scientific outcomes. Nesting material should allow for improved behavioral thermoregulation and thus alleviate this thermal stress. Nesting behavior should change with temperature and material, and the choice between nesting or thermotaxis (movement in response to temperature) should also depend on the balance of these factors, such that mice titrate nesting material against temperature. Naïve CD-1, BALB/c, and C57BL/6 mice (36 male and 36 female/strain in groups of 3) were housed in a set of 2 connected cages, each maintained at a different temperature using a water bath. One cage in each set was 20°C (Nesting cage; NC) while the other was one of 6 temperatures (Temperature cage; TC: 20, 23, 26, 29, 32, or 35°C). The NC contained one of 6 nesting provisions (0, 2, 4, 6, 8, or 10g), changed daily. Food intake and nest scores were measured in both cages. As the difference in temperature between paired cages increased, feed consumption in NC increased. Nesting provision altered differences in nest scores between the 2 paired temperatures. Nest scores in NC increased with increasing provision. In addition, temperature pairings altered the difference in nest scores with the smallest difference between locations at 26°C and 29°C. Mice transferred material from NC to TC but the likelihood of transfer decreased with increasing provision. Overall, mice of different strains and sexes prefer temperatures between 26–29°C and the shift from thermotaxis to nest building is seen between 6 and 10 g of material. Our results suggest that under normal laboratory temperatures, mice should be provided with no less than 6 grams of nesting material, but up to 10 grams may be needed to alleviate thermal distress under typical temperatures.

## Introduction

The *Guide For The Care And Use Of Laboratory Animals* recommends housing rodents, including mice, rats, gerbils, and guinea pigs, at temperatures between 20–26°C [1]. However, in practice, mice are generally housed between 20–24°C [2]. At these temperatures mice eat approximately 60% more than at 30°C in order to meet the energetic needs from increased metabolic demands [3]. This mild thermal stress can alter many aspects of physiology [4] and behavior [5], [6]. These alterations to normal physiology will alter scientific outcomes and has serious implications for animals meant to model human biological systems [7]. Thermal preference research has shown that mice prefer temperatures near 30°C [5], [6], [8] and that thermotaxis (movement in response to temperature) is the primary mode of behavioral thermoregulation in C57BL/6 mice [6]. Preference for temperatures near 30°C is seen for inactive and maintenance behaviors but no preference is seen when active [5], [6]. Thus the temperature preference for one mouse is not constant throughout the day. Warmer temperatures have also been found to increase aggression [9], adding further complication to alleviating thermal discomfort in laboratory mice. Thus, simply increasing laboratory temperatures, as proposed by other authors [7], is not a viable solution, and providing mice with different ambient temperatures within the home cage is impractical in current systems.

In the wild, mice cope with temperature extremes by building nests [10], [11] to minimize heat loss to the environment. Nest building is highly elastic and strongly dependent on the ambient temperature [6], [12], [13]. Providing nesting material for mice to create microclimates within their cage, tailored to their thermal needs, would be an ideal solution to the problem of cold stress. However, with the differences in housing temperatures, humidity levels, and ventilation rates between housing systems, the amount of material needed to alleviate thermal discomfort in particular laboratory settings is unclear. For instance, mice being housed at 26°C (the upper range of recommended temperatures) theoretically would need less material to stay warm than mice being housed at 20°C (the lower range) based on models of heat transfer [7]. A scale that would recommend the amount of nesting material needed to meet a mouse’s thermal needs at various temperatures would be extremely useful for laboratory care staff and researchers.

Ethologists and welfare scientists are often interested in investigating what resources or aspects of the environment are important to captive animals and preference testing can be used as a first step to identifying how an animal perceives the world around it [14], [15]. However, simple preference testing does not indicate how important a preferred resource is to an animal [15], [16], [17]. Motivational paradigms such as consumer demand or behavioral titration can determine an animal’s strength of preference. In particular, a titration experiment varies an unknown commodity against a known one, such as food [16], [18], and establishes the value of the unknown commodity’s worth in terms of the other. Titration is particularly useful when the two behavioral options are ecologically relevant (i.e. they would be balanced by animals in the wild), and when the known commodity can be expressed in terms of objective physical units such as energy or temperature.

The goal of this project was to use the behavioral titration technique to determine how much nesting material is needed to alleviate potential thermal discomfort when mice are housed over a range of ambient temperatures. We hypothesized that location preference, between a warm and cool condition, should change with temperature and amount of material. We predicted that increasing amounts of nesting material would increase nest scores and that nest scores would decrease when mice had access to a warmer ambient temperature. We predicted that mice would spend more time, overall, in temperatures near their lower critical temperature (around 30°C) but this temperature preference would vary depending on the amount of nesting material provided. Previous studies show that ambient temperatures near 30°C are especially preferred when inactive [5], [6], therefore we expected the mice to spend more time inactive in temperatures near 30°C but this too would depend on the amount of nesting material provided. Females are known to prefer slightly warmer temperatures than males [5], [6], therefore we expected the tradeoff between nest and temperature to occur at lower temperatures for males compared to females. We also predicted to see strain preference differences based on temperature and nesting material. Ambient temperature also affects the amount of food eaten in both humans [19], [20] and animals [21], therefore we expected the animals to eat less in warmer cage sets.

## Materials and Methods

Materials and methods were adapted in part from Gaskill et al. [5], [6].

### Animals and Housing

Seventy-two mice from each strain (C57BL/6NCrl; BALB/cAnNCrl; Crl:CD1) arrived at Purdue University, USA from Charles River Laboratories (Wilmington, MA, USA). These three types of mice were chosen because they comprise the most commonly used inbred (C57BL/6NCrl; BALB/cAnNCrl) and outbred (Crl:CD1) research mice. This selection will allow our results to be applicable to the vast majority of the research mouse population. A large difference in body size exists between BALB/c and CD-1 mice at similar ages. Since heat loss is related to the surface area to body weight ratio [22], we decided to control for starting body weight (20–25g) instead of age. Each strain, with the sexes separated, was shipped in two week intervals, to account for the amount of time for testing. Therefore, the age at the start of testing was 6–7 weeks for CD-1s (29.0±5.09g); 11–12 weeks for C57BL/6s (23.8±3.8g); and 13–14 weeks for BALB/c mice (23.8±4.3g). Upon arrival the mice were randomly separated into same sex groups of three and housed in standard laboratory polycarbonate shoebox cages (Alternative Design, Siloam Springs, AR USA; 18.41cm W × 29.21cm D × 12.7cm H) with aspen shaving bedding (Harlan Teklad, Madison, WI USA) and wire cage lids. The mice were kept on a 14:10 Light:Dark photoperiod (lights on at 06:00 AM), at 20°C±1°C with 60±10% relative humidity and given food (Harlan Teklad, Madison, WI USA; Mouse diet 2019) and water *ad libitum*. All housing and procedures associated with this experiment were approved by both Purdue University’s and Charles River’s Institutional Animal Care and Use Committee.

### Thermal Preference Apparatus

**Figure 1:**
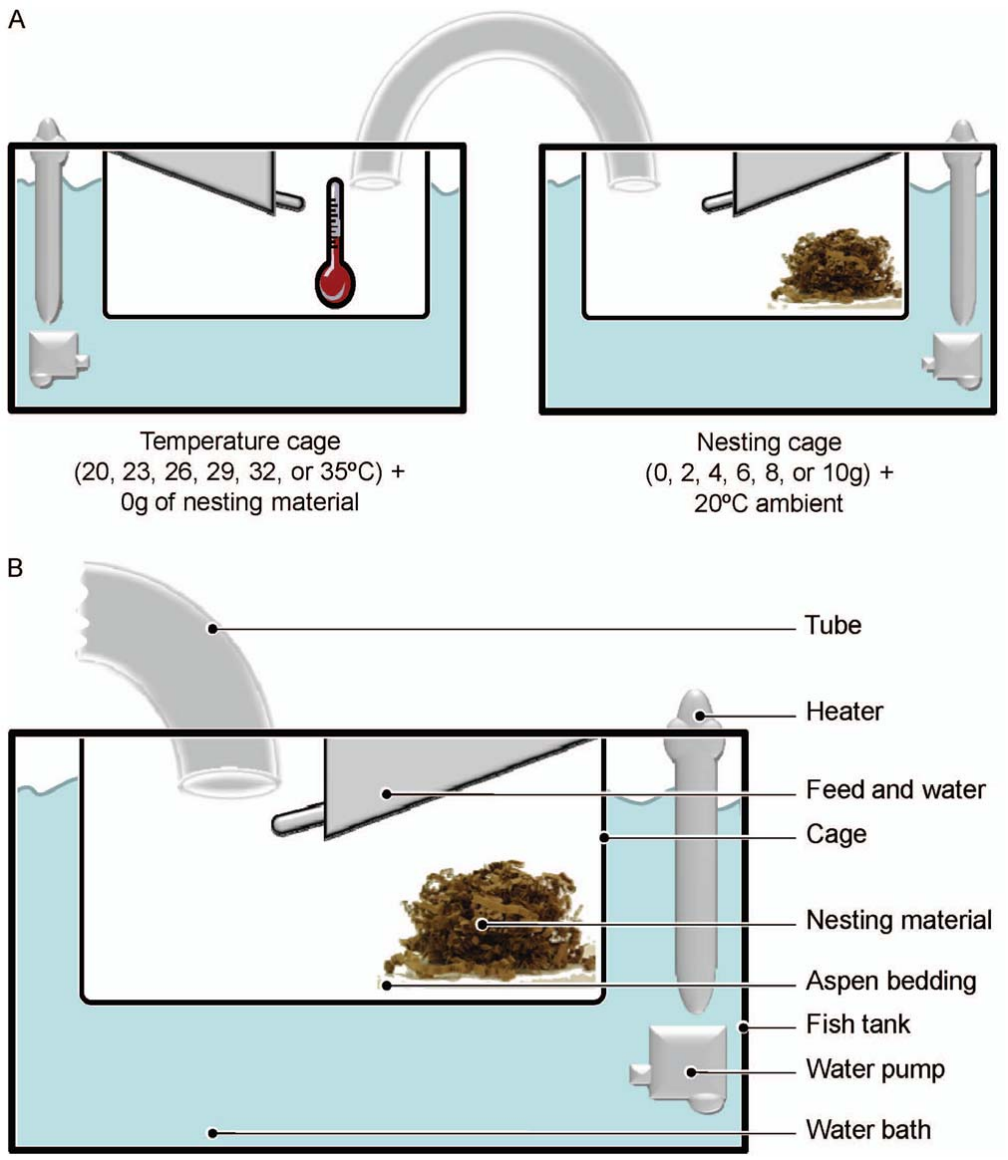
Titration apparatuses. (a) Diagram showing the configuration of water baths and cages for testing cage temperature and nesting material preferences. (b) Diagram depicting elements present in water bath and cage setup. The figures are reproduced with permission from Elsevier [5], [6]

Two 5 gallon glass fish tanks (Figure 1a) were used as water baths, heated by thermostatic electric fish tank heaters, to maintain constant ambient temperatures within the cages (Figure 1b). One cage in each set was 20°C (Nesting cage; NC) while the other was one of six temperatures (Temperature cage; TC): 20°C (a typical laboratory temperature), 23°C, 26°C, 29°C (corresponding to the commonly preferred ambient temperature [23], [24]), 32°C, or 35°C (considered above the thermoneutral zone estimates [2], [23]). Temperatures inside of each cage were confirmed, prior to testing each day of the experiment, by an infra-red thermometer. Submerged cages were of the same make and size as cages in which the mice were housed prior to experimentation and were held in place by the lip of the tank and a thin piece of wood. Approximately 0.64 cm of aspen bedding covered the floor of the cage. Food and water were located on top of all cage lids within the experimental apparatus. Hard plastic hamster tubing (S.A.M., Penn Plax Inc., Hauppauge, NY USA) was used to connect the two cages together through holes in the cage lids. Tube ends were approximately 7.6 cm from the cage floor. Six sets of apparatuses were tested simultaneously (Figure1 a & b).

### Experimental Design

Males and females were tested in alternating weeks, thus the experiment required 12 weeks to complete two replicates. We took precautions to control for position bias and the potential effect of mice in adjacent cages by using visual barriers between cages and by rotating the temperature of the cages each week.

A testing session took 6 days to complete. The day before testing began, mice were trained to use the plastic tubes to transfer back and forth between the two connected cages. On the first day of testing, mice were placed in each cage of the assigned temperature-set twice, alternating every 10 minutes to make sure that the animals experienced both environments and would use the tubes. The mice were all placed in one cage (TC or NC) to begin the day, balanced across the 6 days. The NC contained one of 6 provisions of nesting material (Enviro-dri®; FiberCore, Cleveland, OH, USA): 0, 2, 4, 6, 8, or 10g, changed daily in a balanced design. After each 24 hour period, all nesting material was removed and a new amount was added to NC, so that each group of mice had access to each of the nesting treatments over the course of the testing session. The order of treatment was randomized as a latin square design. Enviro-dri® was chosen as the nesting treatment because it closely resembles materials used in the wild and C57BL/6 mice build better nests with this material than with other options [24]. Nest scores were recorded in both cages based on a 1–5 scale from a previously published protocol [24]. A score of 1 was manipulated material but no central nest cite was evident; 2 was a flat nest; 3 was a cup nest; 4 was an incomplete dome; 5 was a complete and enclosed dome with internal cavity (see [24] for further description of the scoring protocol). On the 7^th^ day, cages within the apparatuses were changed, temperature-sets were rotated and allowed 24 hours to reach the new temperature, while the next group of mice were trained.

### Data Collection

The mice were videotaped continuously over the 6 days for behavioral data collection using infrared cameras and illuminators, digital video recorder and video surveillance software (Inter-Pacific, Wheeling, IL, USA). The location (NC, TC, or Tube) and behavior (Active, Inactive, Maintenance, Nesting, Unknown-in-nest, and Unknown; Table 1) of every mouse was recorded using instantaneous scan samples every 10 min.

**Table 1:** Ethogram of observed behaviors.

Category	Behavior	Description
Active	General locomotion	All locomotive behavior performed on the cage lid, climbing up the cage bars by the food hopper to reach the lid, and locomotion on the floor of the cage.
	Rearing	Seen on the floor of the cage with all an animal’s weight on its hind legs and front legs off the ground. Sniffing movements while on its hind legs were commonly accompanied with this behavior.
	Sniffing	Sniffing was also performed against the cage floor (ground), or in between the bars of the cage lid. Slight upward jerks of the head were seen.
Maintenance	Grooming	All grooming behavior including licking the fur, grooming with the forepaws, and scratching with any limb. Grooming was usually performed in a sitting position with the animal’s hind quarters in contact with the floor.
	Feeding or drinking	The animal would rear up to gnaw at food pellets through the bars of the hopper. The forepaws would usually be used to hold the food pellet steady. The animal would rear up and lick the nipple drinker.
Inactive	Sleeping	The animal was motionless, and either lying curled up on its side, or sitting curled up, with its face tucked into its body and out of sight of the camera. Occasionally interrupted by brief single twitches of the body.
	Still and alert	The animal was sitting or curled up, but in contrast to sleep, the face was lifted. The animal either sat motionless, or would appear to be orientating its head to sounds outside of the cage.
	Inactive in nest	The animal within the nest, due to camera angles, cannot clearly be seen but no movement within the nest can be detected. It is assumed that the animal is sleeping within the nest. This is distinguishable from other behaviors within the nest because movement within the nest or of the nest itself is not observed.
Nesting	Pull in	Characterized by the animal reaching out of the nest and pulling sawdust or nesting material to the edge of the nest. The animal may also grasp the material in its mouth and drag it into the nest site. Gathering is distinct from locomotion in that the hind legs do not leave the nest site, and each time the animal reaches out of the nest it pulls its forelegs back in.
	Carrying	Locomotion with material, such as large pieces of bedding or nesting material in the mouth.
	Fraying	The animal uses sideways movement of the forepaws to draw material through the beak. Gnawing movements of the jaw and jerking movements with the head are also seen. As a result the edges of the nesting material are bitten off or large pieces of bedding are split into smaller fibers.
	Push-Dig	The forward pushing and kicking of substrate material with fast alternating movements with the forepaws often combined with forward locomotion.
	Sorting	The deliberate action of placing specific nesting material strips or bedding material into a particular location while sitting within the nest site.
	Digging	Removing, or apparently trying to remove, substrate material from a certain place by series of fast alternating movement of the forepaws, as a consequence of which the material heaps up under the abdomen of the animal.
	Scrape-dig	The series of forepaw movements are alternated by a few hindwards kicking movements of both hind legs simultaneously, through which the heap under the abdomen of the animal is transported further backwards.
	Fluffing	An unseen nesting behavior, due to insufficient camera angles or view from inside the nest, which results in the enlargement of the nest from the inside. Walls of the nest will appear to jump and the nest as a whole will enlarge. It is assumed that the animal is hollowing out the inside of the nest by pushing the walls back and up.
Unknown in Nest	Unknown	An animal is inside of the nest but unsure of the behavior being occurring inside of the nest. This is different from Fluffing in that the nest does not appear to be growing or occurs out of the sequence on nest building. This is also different from Inactive in Nest in that movement is seen within the nest.
Unknown	Unknown	An animal’s behavior cannot be determined or the view of the animal is blocked while in or outside of the nest.

#### Food consumption

Food consumption was measured before and after each 6 day testing session from both adjoined cages.

#### Nest scores

Nest scores were recorded daily from both NC and TC at the end of the 24 hour test period, before nesting treatments were changed. Nest scores were recorded from both cages because mice will attempt to build a simple nest out of bedding material when other substrate is not provided. To compare nest scores between NC and TC, the nest score from TC was subtracted from NC to get the difference in nest score between the two cages.

#### Behavior

Population time budgets were calculated for each group of mice by counting the total number of times each category of behavior was observed in each location (i.e. NC, TC, and the tube) for each day and dividing this count by the total number of observations for that group. Following this calculation, data from the tube and unknown behaviors were excluded from the analysis. The percent observations from NC (plus the smallest observation in order to avoid zero values) were divided by TC (again plus the smallest observation). The log of this value was taken in order to normalize the ratio of observations in NC relative to TC.

### Analysis

Behavior, nest score, and food consumption analyses were performed as split-plot ANOVA using GLM, in JMP 6 for Windows. The assumptions of GLM (normality of error, homogeneity of variance, and linearity) were confirmed post-hoc, and appropriate transformations were made to meet these assumptions [25]. Significant effects were then analyzed using post-hoc Bonferroni corrected planned comparisons, or custom contrasts in JMP. ‘Test slices’ or Tukey tests in JMP were used to identify behaviors where significant differences were found. t-tests were then used to confirm, post hoc, that the NC:TC ratio was significantly different from zero.

To avoid pseudoreplication and accommodate repeated measures, analyses were blocked by Group of mice, nested within Strain, Sex, and Temperature-Set. Group of mice cannot be treated as a random effect (there is not a meaningful wider population of groups of three mice representing unique and indivisible components of variance from which we selected our groups of three mice, and to which our results could pertain) [26], and was therefore treated as fixed (i.e. as a split plot). Any observations of mice in the tube and the unknown behavior category were eliminated from the dataset. Thus the behavioral time budget does not total 100% and the independent variables are not co-linear. In essence change in one behavioral category will not directly influence the level of another behavioral category. Some mice were found to carry nesting material from the NC to the TC, therefore the variable Carry over was added to the nest score and behavior analysis. In addition, a binary logistic regression in JMP was run to determine the likelihood ratios of when mice were more likely to transfer material.

## Results

### Body Weight

The difference in bodyweight before and after the experiment was documented but no statistical differences due to temperature or nesting material were seen. The average body weight by each type of mouse at the beginning of the experiment was as follows: C57BL/6 Females = 20.6g; Males = 27.1g; BALB/c Females = 19.8g; Males = 27.7g; CD-1 Females = 25.5g; Males = 32.6g. Bodyweight at the end of the experiment was: C57BL/6 Females = 20.7g; Males = 27.1g; BALB/c Females = 20.3g; Males = 27.8g; CD-1 Females = 25.6g; Males = 34.4g.

### Food Consumption

We first predicted that cage sets with warmer temperatures would result in a reduction in the amount of food consumed. The overall amount of food consumed was not significantly altered in any of the temperature-sets (GLM: F_5,71_ = 0.43; P = 0.82). However, there was a significant interaction between temperature-set and the location (TC or NC) where they consumed the food (GLM: F_5,71_ = 2.91; P = 0.019; Figure S1). A decrease in food consumption with increasing TC temperatures was found (Linear Contrast: F_1,71_ = 8.65; P = 0.004) but no significant trend was found in NC (Linear Contrast: F_1,71_ = 1.36; P = 0.24). The linear contrast in TC was also found to be significantly different from the one in NC (Contrast: F_1,71_ = 8.45; P = 0.004).

### Nest Scores

Strain and Temperature-Set was found to alter nest scores (GLM: F_10,308_ = 2.76; P = 0.003). In the 20–20°C temperature-set, C57BL/6 and CD-1 mice built better nests in NC (t _α/18_; P<0.05), but no significant differences in nest building between the two locations was found for BALB/c mice (t _α/18_; P>0.05). No differences in nest building were found in the 23, 26, or 29°C temperature-sets for any of the strains (t _α/18_; P>0.05). BALB/c and CD-1 mice built significantly better nests in NC at 32°C (t _α/18_; P<0.05), but this pattern was not shown in C57BL/6 mice (t _α/18_; P>0.05). In the warmest temperature-set, 35°C, all the stains built significantly better nests in NC (t _α/18_; P<0.05). 

Nest quality was altered by interactions between Strain and Sex (GLM: F_2,308_ = 6.76; P = 0.001). Female BALB/c mice built significantly better nests in NC (t _α/6_; P<0.05), but the other two strains showed no building difference between the two locations (t _α/6_; P>0.05). No differences in nest building for females were found between the strains (Tukey: P>0.05). Male C57BL/6s and CD-1s built better nests in NC (t _α/6_; P<0.05) but the BALB/c mice showed no differences in location (t _α/6_; P>0.05). Male CD-1s built significantly better nests in NC than BALB/c males (Tukey: P<0.05), but BALB/c and C57BL/6 male’s building was not significantly different from one another (Tukey: P>0.05). CD-1 and C57BL/6 males built significantly better nests in NC compared to females of their respective strain (Tukey: P<0.05). However, no significant differences were found between male and female BALB/c mice (Tukey: P>0.05).

#### Carryover influence

**Figure 2:**
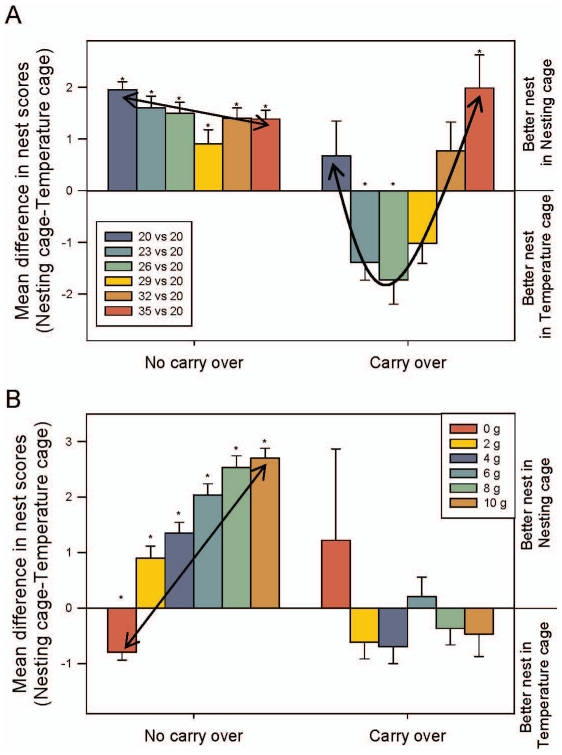
The mean difference in nest score values between the nesting cage and the temperature cage. Nest scores partitioned by occurrences of nesting material carryover by (a) cage sets and (b) amount of material provided. A negative value indicates a better nest built in the temperature cage and a positive value indicates a better nest in the nesting cage. LSM and SE are plotted and significant t-tests (value different from zero; α corrected for the number of comparisons) are indicated by asterisks. A diagonal line indicates a significant linear trend and a curved line indicates a significant quadratic trend.

Nest quality was also affected by temperature (GLM: F_5,308_ = 12.6; P<0.001), but nest scores changed when the mice transferred nesting material (GLM: F_5,308_ = 6.6; P<0.001; Figure 2a). Mice that did not transfer the material show a transitive decrease in nest score with temperature (Linear Contrast: F_1,308_ = 7.21; P = 0.007), with the highest nest score found in the 20–20°C temperature-sets. All nest scores were significantly higher in NC at all temperatures (t _α/12_; P<0.05). When material is transferred, a significant quadratic trend was found (Quadratic Contrast: F_1,308_ = 32.1; P<0.001). Here nest scores were significantly higher in TC at 23 and 26°C and NC at 35°C (t _α/12;_P<0.05). All other temperatures showed no significant differences in nest scores between the two cages.

As predicted a significant main effect of nesting material amount on nest quality was found (GLM: F_5,308_ = 7.53; P<0.001). However, if mice transferred nesting material from NC to TC this significantly altered the difference in nest quality at different amounts (GLM: F_5,308_ = 3.9; P = 0.002; Figure 2b). When the nesting material was not transferred, nest scores increased with an increasing amount of nesting material (Linear Contrast: F_1,308_ =  244.5; P<0.001). Overall mice built better nests in NC but when they received the control nesting treatment of 0g, they built a better nest in TC (t _α/12_; P<0.05). However, when the mice transferred the material, there was no linear trend (Linear Contrast: F_1,308_ =  0.52; P = 0.47) and no significant differences in nest scores between the two locations were found (t _α/12_; P>0.05).

The sexes also showed a disparity in building location when material was transferred (GLM: F_2,308_ = 8.07; P = 0.005; Figure S2a). When the material remained in NC, both sexes built significantly better nests in NC (t _α/4;_ P>0.05) and were not different from one another (Tukey: P>0.05). However, when material was moved, females built significantly better nests in TC (t _α/4;_ P<0.05) but males showed no difference in nest building between the two locations (t _α/4;_ P>0.05). However, nest building was significantly different between the two sexes when material was transferred (Tukey: P<0.05).

The three strains also showed differences in nest building when material was transferred (GLM: F_2,308_ = 12.6; P<0.001; Figure S2b). When the material remained in NC, all strains built significantly better nests in NC instead of TC (t _α/6;_P<0.05) and C57BL/6 mice built the lowest quality nests in NC compared to the other two strains (Tukey: P<0.05). However, when material was transferred, C57BL/6s built a better nest in NC but was not significantly different from TC (t _α/6;_P>0.05). BALB/c mice built a significantly better nest in TC (t _α/6;_P<0.05) which was significantly different from C57BL/6s (Tukey: P<0.05). CD-1s showed no difference in building between the two strains (Tukey: P>0.05) or the two locations (t _α/6;_P>0.05).

#### Likelihood of carryover

**Figure 3:**
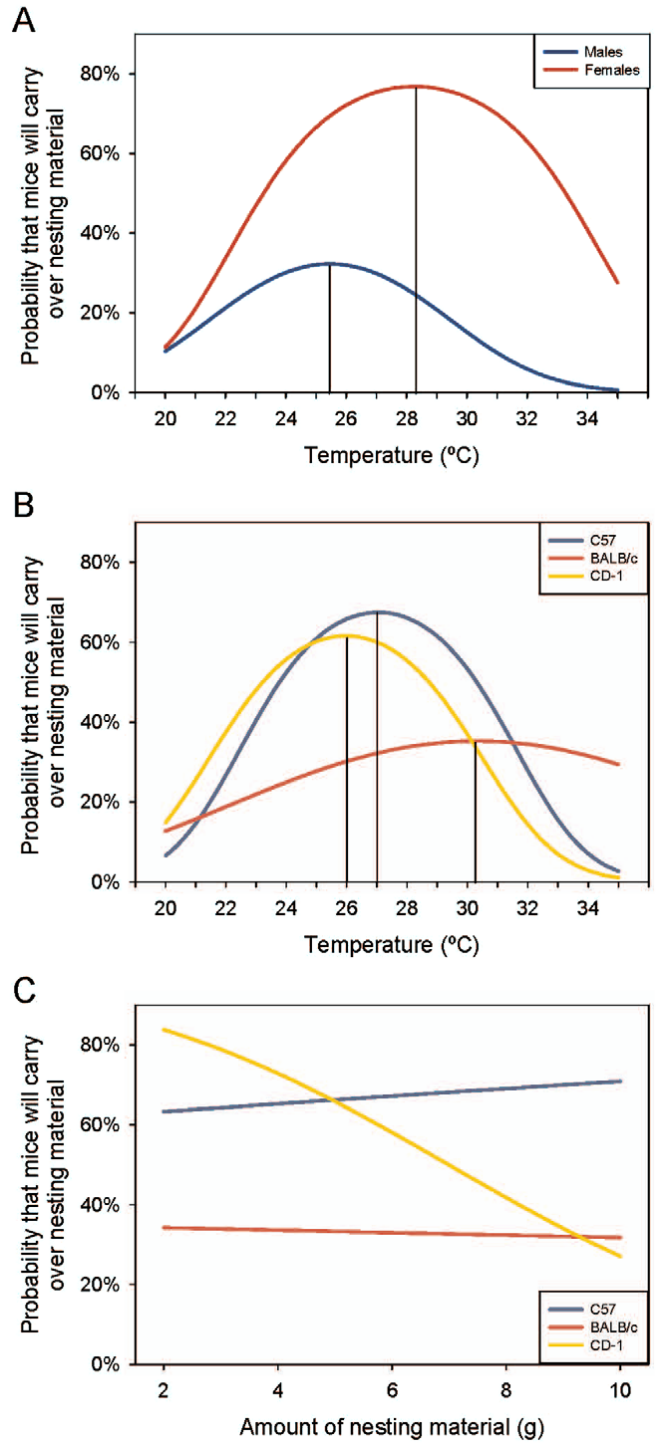
Likelihood of nesting material being transferred to the temperature cage. Data is plotted by (a) sex and temperature; (b) strain and temperature and; (c) amount of nesting material and strain. Quadratic peaks are indicated by solid vertical lines.

The transfer of nesting material from the NC to TC was an unexpected observation in this experiment. There was a significant Sex effect: females were more likely to transfer material than males (LR χ^2^ = 56.4; P<0.001; Figure 3a). In addition, the temperature at the peak likelihood of carryover for females (≈28°C) was higher than males (≈25°C) (LR χ^2^ = 15.70; P<0.001). The likelihood of different strains to carry over material was also affected by temperature (LR χ^2^ = 12.43; P = 0.002; Figure 3b). C57BL/6 mice carried over the most often, peaking at 70% likelihood at approximately 27°C. The likelihood of carryover for CD-1 mice peaked at 60% at approximately 27°C and BALB/c mice at 35% at approximately 30°C. The likelihood of material transfer was significantly different between BALB/c and C57BL/6 mice (Custom test: _α/3:_ χ^2^ = 8.89; P = 0.002) but not for CD-1 mice (Custom test: _α/3:_ χ^2^ = 4.57; P = 0.03). The temperature at which the peak likelihood of carryover occurred for BALB/c (≈30°C) was higher than CD-1 mice (≈26°C) (Custom test: _α/3:_ χ^2^ = 12.3; P<0.001). No significant differences between C57BL/6 mice and the other two strains for peak likelihood were found. The amount of material provided also significantly affected the likelihood of the strains carrying over the nesting material (LR χ^2^ = 10.70; P = 0.005; Figure 3c). CD-1’s showed a peak likelihood of 80%, which decreased as provision of nesting material increased (Custom test: _α/3:_ P <0.001). The slope of the line for C57BL/6s (Custom test: _α/3:_ χ^2^ = 0.26;P<0.61) and BALB/cs (Custom test: _α/3:_ χ^2^ = 0.3; P = 0.86) was not significantly different from zero.

### Location and Behavior

#### Temperature-Set effects

Some unpredicted main effects were found based on where the mouse strains spent their time (GLM: F_2,2425_ = 78.41; P<0.001). BALB/c mice spent more time in NC than the other two strains (Tukey: P<0.05). While CD-1s still spent the majority of their time in NC, this amount of time was significantly less than the BALB/c mice (Tukey: P<0.05). C57BL/6 mice were the only strain to spend the majority of their time in TC (Tukey: P<0.05). The sexes also showed differences in their location preferences (GLM: F_1,2425_ = 120.4; P<0.001). Overall males spent more time in NC while females spent more time in the TC.

**Figure 4:**
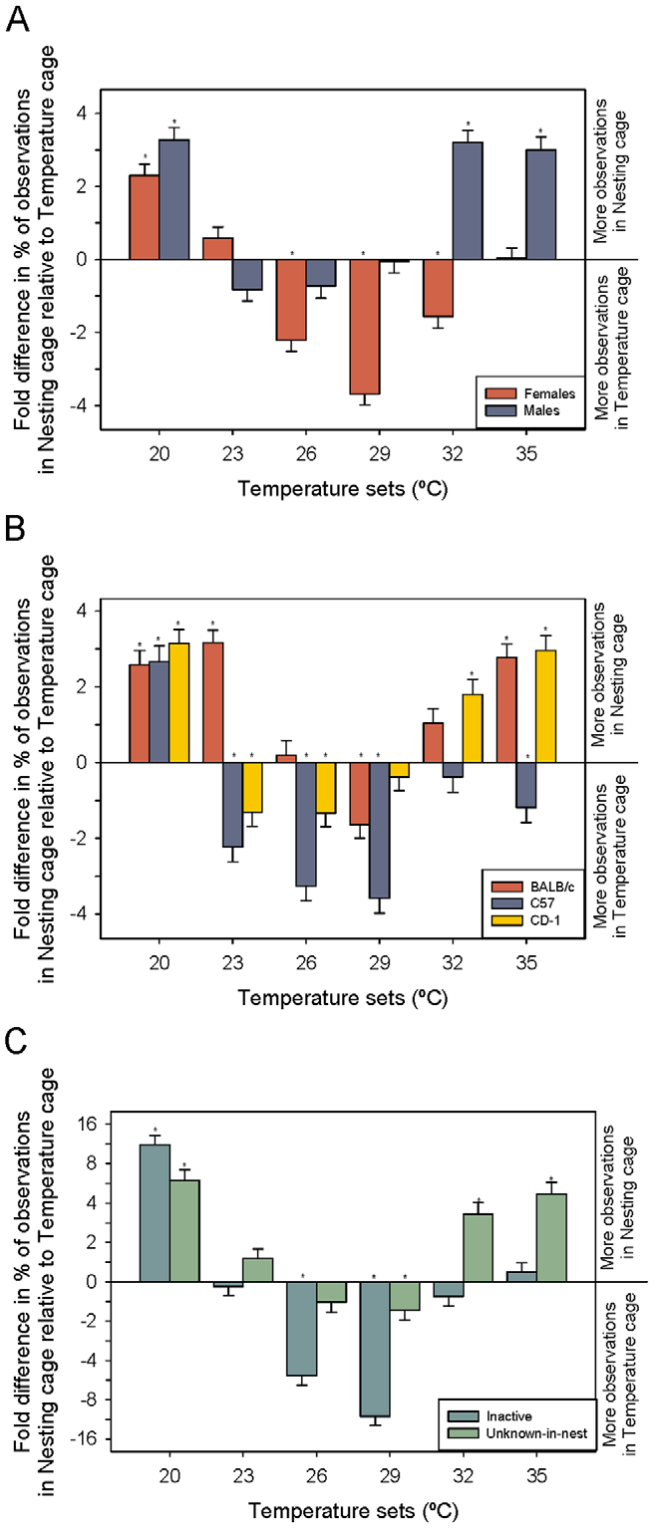
Location preference due to temperature-set. Fold difference in percent of location observations between the nesting cage relative to the temperature cage. Effects of temperature-set are plotted by interactions with (a) sex; (b) strain and; (c) behavior. LSM and SE are plotted and significant t-tests (value different from zero-α corrected for the number of comparisons) are indicated by asterisks.

We predicted that the temperature a cool cage was paired with would affect the preference for nesting material. As predicted Temperature-Set affected preference but depended on Sex (GLM: F_5,2425_ = 25.6; P<0.001; Figure 4a). Males significantly preferred NC over TC at 20, 32, and 35°C but preferences were equal in the middle three temperatures (t _α/12_; P<0.05). Females preferred NC at 20°C but TC at 26, 29, and 32°C (t _α/12_; P<0.05). No difference from zero was found at 35°C.

Preference differences were also seen between the different strains (GLM: F_10,2425_ = 13.1; P<0.001; Figure 4b). BALB/c mice preferred NC at 20, 23, and 35°C but only preferred TC at 29°C (t _α/18_; P<0.05). C57BL/6s spent significantly more time in TC at 23, 26, 29, and 35°C. NC was preferred over TC only at 20°C (t _α/18_; P<0.05). CD-1s preferred NC at 20, 32, and 35°C and TC at 23 and 26°C (t _α/18_; P<0.05).

Behavior was also altered based on temperature-set (GLM: F_20,2425_ = 18.2; P<0.001; Figure 4c). Test slices identified inactive and unknown-in-nest as the only behaviors with differences due to temperature. As predicted, inactive behavior was seen more often in TC at 29°C, which is near their preferred temperature of 30°C. However, a significant amount of inactivity was also seen in TC at 26°C and in NC at 20°C (t _α/6_; P<0.05). All other temperatures showed equal amounts of inactivity in both locations. Significantly more unknown-in-nest behaviors were seen in NC at 20, 32, and 35°C (t _α/6_; P<0.05) and in TC at 29°C (t _α/6_; P<0.05). Equal amounts of unknown-in-nest behavior were seen at 23 and 26°C.

**Figure 5:**
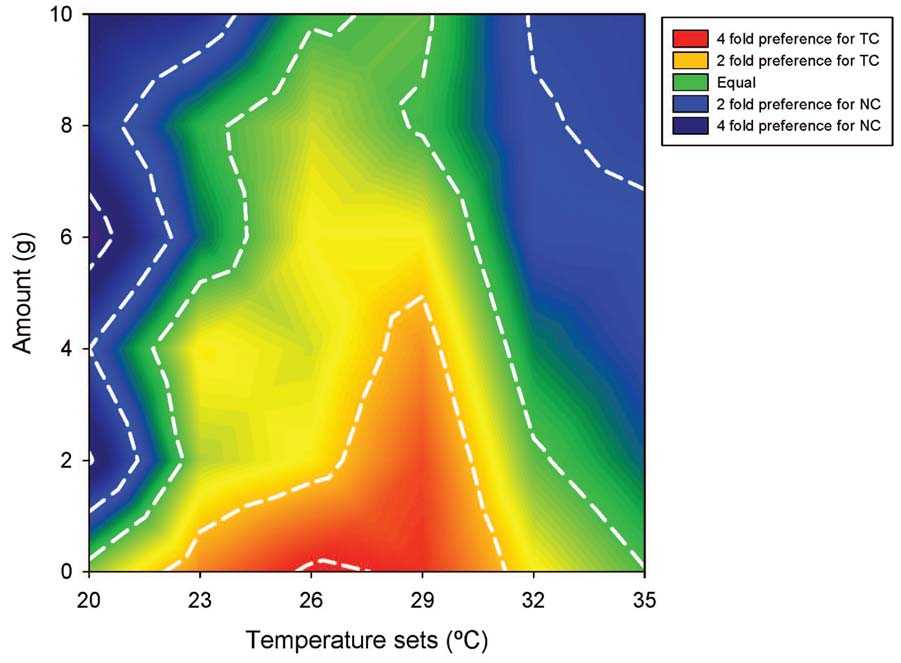
Location preference by titrated variables. Mean difference in percent of observations between the nesting cage and the temperature cage for the temperature-set by amount of nesting material interaction. The green area indicates equal preference for NC and TC. Blue and purple shading indicate a 2 and 4 fold preference for NC. Orange and red shading indicate 2 and 4 fold preferences for TC.

A significant interaction between Temperature-set and Amount of nesting material was found (GLM: F_25,2425_ = 2.54; P<0.001; Figure 5). At 20°C, NC was preferred at all amounts of nesting material except 0 grams (t _α/36_; P<0.05). At 23°C, NC was only preferred at 10 grams and TC was preferred at 0 grams (t _α/36_; P<0.05). Significantly more time was spent in TC with 0 grams at 26°C (t _α/36_; P<0.05). At 29°C, TC was significantly preferred when mice were given 0, 2, and 4 grams of nesting material (t _α/6_; P<0.05). Equal preferences were seen at all other temperatures. At 32°C, significantly more time was spent in NC with 8 and 10 grams (t _α/36_; P<0.05). At the warmest temperature, 35°C, NC was preferred with 4–10 grams and 0 and 2 grams showed no differences (t _α/6_; P<0.05).

#### Amount of nesting material effects

**Figure 6:**
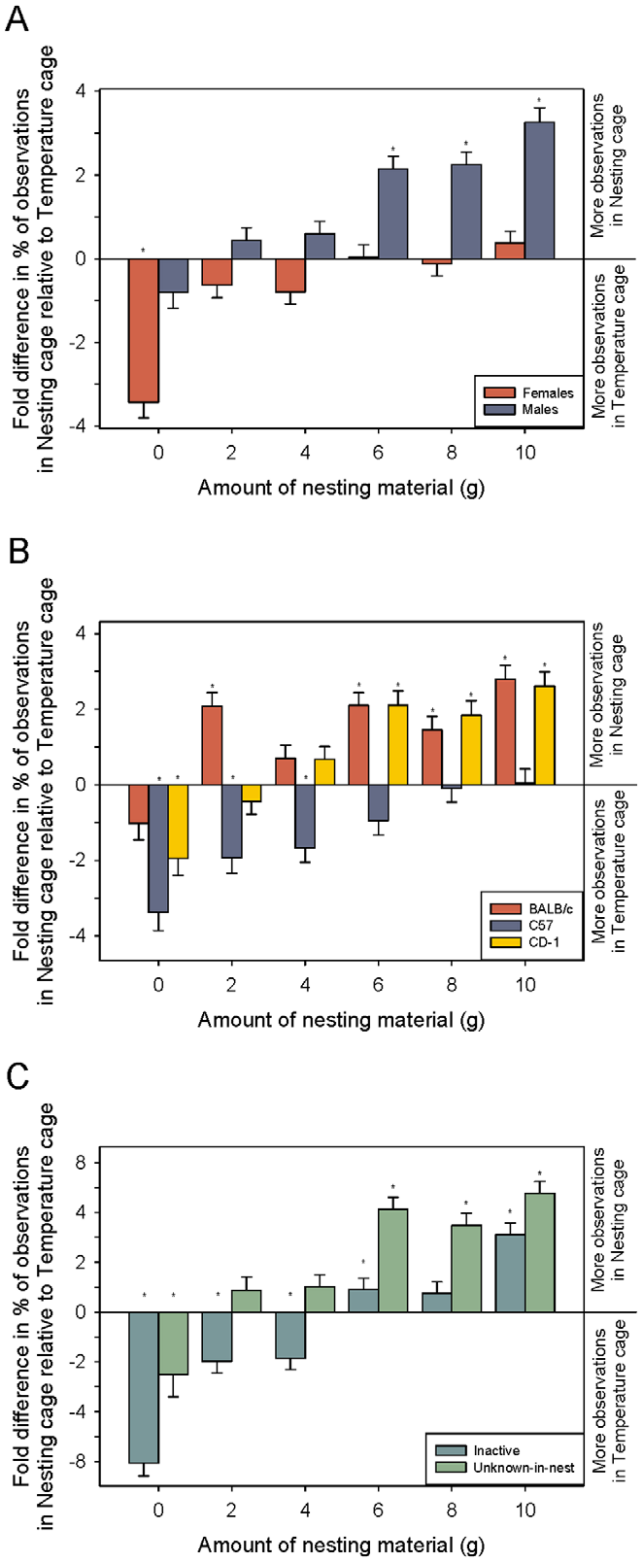
Location preference due to amount of nesting material. Fold difference in percent of observations between the nesting cage relative to the temperature cage. Effects of the amount of nesting material provided are plotted by interactions with (a) sex; (b) strain and; (c) behavior. LSM and SE are plotted and significant t-tests (value different from zero-α corrected for the number of comparisons) are indicated by asterisks.

As predicted, a significant interaction between the amount of nesting material provided and sex was found (GLM: F_5,2425_ = 5.95; P = 0.019; Figure 6a). Post-hoc t-tests showed that females spent significantly more time in the TC than NC when no material was provided but spent equal time in both TC and NC for all other amounts. Males showed that with increasing amount of material there was an increasing amount of time spent in NC (Linear Contrast: F_1,2425_ = 65.3; P<0.001). Significantly more time was found to be spent in NC at 6, 8, and 10 grams of nesting material (t _α/12_; P<0.05). 

Where mice preferred to spend their time was also significantly affected by amount and strain (GLM: F_10,2425_ = 2.77; P = 0.002; Figure 6b). BALB/c mice spent significantly more time in NC with 2, 6, 8, and 10 grams of nesting material (t _α/18_; P<0.05). The time spent in either cage was not significantly different for the other two amounts (0 and 4 grams). C57BL/6s spent significantly more time in TC when given 0, 2, or 4 grams material (t _α/18_; P<0.05). No differences were seen for the other three amounts. CD-1s spent significantly more time in TC with 0 grams, but when given 6, 8, or 10 grams they spent more time in NC (t _α/18_; P<0.05).

Differences in behavior were also seen depending on the amount of material provided (GLM: F_20,2425_ = 8.2; P<0.001; Figure 6c). Test slices in JMP identified inactive and unknown-in-nest as the only behaviors with differences due to amount. Mice preferred to be inactive in TC when provided 0, 2, and 4 grams of material but preferred NC with 6 or 10 grams (t _α/6_; P<0.05). Unknown-in-nest behaviors were observed more often in TC when no material was provided but were observed in NC when given 6, 8, or 10 grams (t _α/18_; P<0.05).

Other behavioral observation differences were affected by the main effect of sex (GLM: F_4,2425_ = 36.5; P<0.001; Figure S3a). Test slices in JMP identified inactive and unknown-in-nest as the only behaviors with differences due to temperature. Females were significantly more inactive in TC while males were significantly more inactive in NC (t _α/10_; P<0.05). Males also spent significantly more time nest building and unknown-in-nest in NC (t _α/10_; P<0.05), while females showed no preferences.

Strain was also found to affect the location of behavior (GLM: F_8,2425_ = 16.7; P<0.001; Figure S3b). Test slices in JMP identified inactive, maintenance, and unknown-in-nest as the only behaviors with differences due to temperature. BALB/c mice spent significantly more time inactive in NC, while C57BL/6 mice spent more time in TC (t _α/9_; P<0.05). C57BL/6s spend significantly more time in TC for maintenance behaviors but the other two strains showed no differences (t _α/9_; P<0.05). Unknown-in-nest behaviors were observed significantly often in NC by BALB/c and CD-1 mice (t _α/9_; P<0.05).

## Discussion

This experiment shows for the first time the preference tradeoff between temperature and nesting material, based on the combination of the two factors. The knowledge of this tradeoff is extremely important because not all laboratory temperatures are identical, and therefore it is unknown how much material is needed to eliminate mouse thermal discomfort under various conditions. Furthermore, nesting material is increasingly being implemented in the United States and is considered a standard husbandry item in Europe [27]. Therefore, this information can be applied by laboratory managers and researchers to determine the appropriate provision of material depending on the conditions of their facility.

The effect of warmer or cooler temperatures on food consumption has been documented in humans [19], [20] as well as other animals [21]. Generally, increasing temperatures result in a reduction in food intake. In this experiment, regardless of the combination of temperatures, we observed no significant differences in overall feed intake. However, a linear decrease in food eaten in TC was found but not in NC. It appears that the temperature in their immediate surroundings had an effect on food consumption but was ultimately balanced out between the two locations. It was surprising that no linear increase was observed in NC to counteract less food being eaten in the warmer TC cages. The most likely reason for this lack of differences in Temperature-sets was because the animals were periodically exposed to the cooler temperatures in NC. This constant flux of temperature exposure did not allow their bodies to acclimate, thus resulting in no overall changes in food consumed.

Our mice showed the expected nest building responses to both temperature and amount of nesting material, when material was found solely in NC. However, when the mice transferred the material, better nests were no longer consistently built in NC. This decision to carry over nesting material from one cage to another was a surprising result, as a similar experiment by Gaskill et al [6] did not encounter this behavior. However, this transferring and combining of resources has been documented in other experiments [28], [29], [30]. Transferring the material was generally performed 1–2 strands at a time, and required a substantial amount of time and effort from the mice. While this is not a direct measure of motivation, it does convey their willingness to work in order to achieve a combination of material and temperature. The act of combining these resources points to a preference for temperature or thermotaxis as the predominant mode of behavioral thermoregulation when the likelihood of carryover is high. C57BL/6 and female mice are highly likely to transfer material to TC and spend the majority of their time in that location. This mode of behavioral thermoregulation in C57BL/6 mice is supported by previous research [6]. On the other hand, BALB/c mice show an overall low likelihood of material transfer and consequently spend the majority of their time in NC. This suggests that nest building is the primary mode of behavioral thermoregulation for BALB/c mice. If true, it stands to reason that they would have low motivation to transfer material to the other temperatures.

CD-1 mice on the other hand, employ a different strategy than the other two strains. They appear to tradeoff between nest building and thermotaxis based on the amount of nesting material provided or temperature. However, the provision of nesting material seems to be the main factor they are basing this decision on. When material provision is low, they show high motivation to transfer material and combine it with temperature, or use simple thermotaxis. They do not begin to spend significantly more time in NC until 6 grams or more of nesting material was provided. Most likely this is the smallest amount of material that can be used to build a suitably insulating nest. Consequently, the motivation to transfer material also declines as the provision of material increases. The probability of material transfer for the CD-1 mice ranges from a level similar to a strain utilizing thermotaxis (C57BL/6) when there is a small amount of material, to a level similar to the nest building strain (BALB/c) as nesting provision increases. While CD-1 mice may switch strategies, they seem to favor nest building over thermotaxis.

It is possible that the reason CD-1 mice employ a strategy different from the other two strains is due to their young age or exposure to enrichment at an earlier age. Thermal preference studies have found differences in temperature selection due to age [31], [32], but preferences are consistently near 30°C in older mice (3–11 months [8], [31]). Indeed other studies have found that exposure to enrichments at a younger age is more impactful than at an older age when preferences and habits have developed [33], [34]. Therefore animals exposed to this enrichment at a younger age may more effectively utilize the enrichment [35].

If the probability of material transfer can be used to indicate the primary mode of behavioral thermoregulation, then it is interesting that this behavior was seen in all combinations of mice and environmental variables. It appears that mice retain some underlying motivation for nesting material, even if their thermal needs are met. It is likely the drive to build a nest may serve a purpose other than thermoregulation [6], [36]. Shelters or retreat spaces have been shown to decrease stress and fearfulness in laboratory animals [36], [37].

Previous preference work points to slight differences in thermal preference between the sexes as well as differences in nest shape as temperatures increase [5], [6]. Gaskill et al [6] found that female C57BL/6 mice built better, more dome-like, nests at both 25°C and 30°C, perhaps indicating a sustained thermal challenge even at these higher temperatures. These data should be extrapolated to the other strains, with caution, due to differences in behavioral thermoregulation (found in this study), genetic background [38], and the fact that mice were not properly acclimated to these temperatures. Nonetheless, behavioral location data from this study support this idea as females showed a preference for TC from 23 up to 32°C and equal preference for both locations at 35°C. This lack of preference at 35°C was surprising as this temperature was meant to be experienced as too warm by the mice and slightly aversive. This suggests that females generally do not find this temperature as aversive as previously thought and is preferred equally to an average amount of nesting material. Males on the other hand, show no preferences in the middle temperatures (23, 26, and 29°C). Therefore, the average amount of nesting material and these temperatures are also perceived as equal. Based on these results, it appears that the preferences for males (averaged over all the strains) are skewed slightly toward cooler temperatures (between 23 and 26°C) and females toward warmer temperatures (≈29°C). While some differences in thermoregulation are seen due to sex hormones [39] these differences are likely attributed to simple differences in body weight [7]. Regardless of the mechanism influencing temperature preference, the existence of these differences further emphasizes that there is no one perfect temperature for laboratory mice [5], [6].

Although we have shown thermal disparity between the sexes, it appears that 20°C is a universally cool temperature for both sexes and all strains [6]. At this temperature both genders and all strains spent significantly more time in NC than TC. The highest quality nest building is also seen at this temperature indicating some degree of thermal discomfort [40], [41]. This is not surprising as metabolism is increased from the basal metabolic rate [2] and impaired immune function [4] has been shown at this temperature. 

The behavioral ethogram covered 6 categories of behavior but only a few showed any significant differences due to our treatments. Previous studies suggest that temperature or nesting material may be more essential to mice while inactive [5], [6]. This is not surprising, because the smallest amount of heat is produced by the body at this time [42], thus the resting basal metabolic rate [2] and body temperature decreases [43]. Because less heat is produced, and assuming the same rate of heat loss to the environment is occurring, the animal needs to utilize other behavioral measures to minimize heat loss. This usually includes altering the amount of surface area exposed to the environment [22]. Curling into a ball, huddling with conspecifics, or nest building can help accomplish this goal. While not directly measured, it was observed that mice would huddle together in cooler temperatures. In warmer temperatures, on the other hand, mice would attempt to huddle but the huddle would not last long. The mice would eventually move away from one another, lying elongated instead of in a hunched position. Rat pup huddles have been documented to engage in this group alteration of exposed surface area [44]. All strains and sexes show preferences to sleep in temperatures between 26–29°C and appear to be indifferent at higher temperatures. The unknown-in-nest observations in NC insinuate that some divergence in preference between strains and sexes occurs at 32 and 35°C. This behavioral category illustrates that the animals are occupying a large enough nest that they cannot be directly observed. Thus, some animals found the cooler cage with nesting material more preferable than the warm temperature, resulting in nearly equal observations in both locations.

A practical question, especially from an economic standpoint, is how much material is needed to alleviate any thermal distress and how much does that amount change under standard laboratory temperatures. Other experiments have investigated the amount of material collected by mice [12], [45] and what kinds of material to give them [24], [28], [30], but the authors know of no studies that measured how much is needed from the animals perspective to alleviate thermal discomfort. During inactivity in this study, temperature was chosen over nesting material until 6 grams was provided. This leads us to believe that over the three strains, at least 6 grams is needed to build a sufficient nest. Therefore, no less than 6 grams should be given to mice at any recommended temperature (20–26°C [1]). However, for the temperatures within this range, mice saw all nesting amounts (except our control of 0g) as equal to temperature and only significantly selected NC once 10 grams was provided. Therefore, we recommend providing as much as 10 grams in non-ventilated caging. It is possible that more nesting material may be needed for ventilated caging because of the increase in convective heat loss. However, more research is needed before a recommendation for that type of housing can be proposed. Providing mice with too much material is not likely to be detrimental to them. The beauty of this type of enrichment is that it provides the animals with control over their microenvironment, allowing them to build a nest according to their specific needs.

Our location preference results, which do not incorporate specific behaviors, may have been slightly lowered based on the way that the data was processed. Location means were averaged over every behavioral category in our ethogram. Since mice prefer different temperatures at different parts of the day as well as for different behaviors, this analysis controls for the particular behaviors that drive these preferences, such as inactive behavior. Therefore more frequent behaviors are weighted equally with other less frequently observed behaviors, such as nest building. Thus, location preference values are the mean location preference for all behaviors.

## Supporting Information

Figure S1 **Total feed consumption.** Consumption averaged by temperature-set, from either the nesting cage or temperature cage over the six day testing period. LSM and SE are plotted and the diagonal line indicates a significant linear contrast.
(TIF)

Figure S2 **The mean difference in nest score values between the nesting cage and the temperature cage.** Nest scores partitioned by occurrences of nesting material carryover by (a) sex; and (b) strain. A negative value indicates a better nest built in the temperature cage and a positive value indicates a better nest in the nesting cage. LSM and SE are plotted and significant Bonferroni corrected planned comparisons are indicated by †.
(TIF)

Figure S3 **Location preference by behavior.** Differences in behavior are plotted by interactions with (a) sex and (b) strain. LSM and SE are plotted and significant t-tests (value different from zero-α corrected for the number of comparisons) are indicated by asterisks.
(TIF)
