# Supplementary figures and images for: Heat or Insulation: Behavioral Titration of Mouse Preference for Warmth or Access to a Nest
Source: PLoS One. 2012 Mar 30;7(3):e32799. doi: 10.1371/journal.pone.0032799 (PMC3316552; doi:10.1371/journal.pone.0032799)

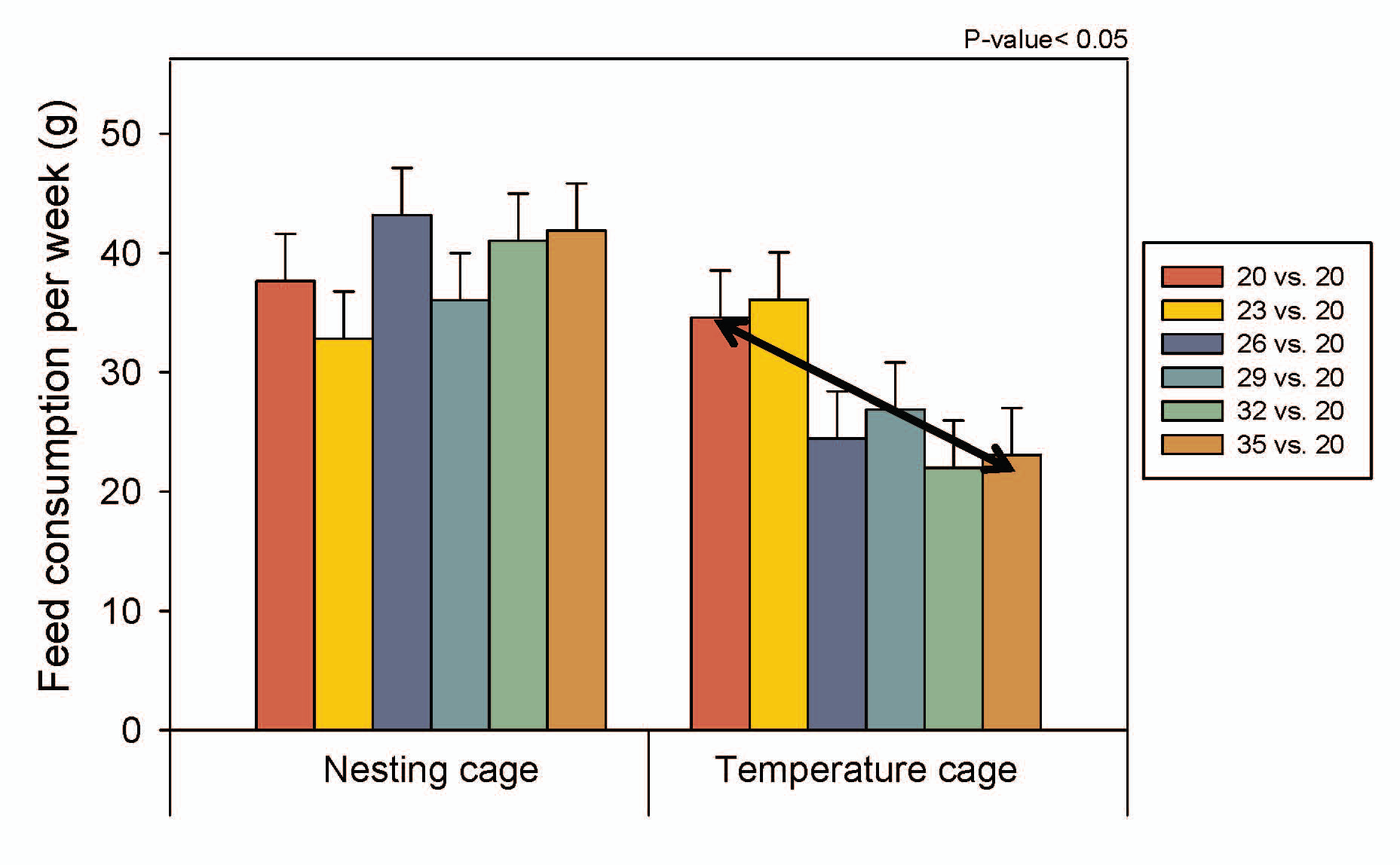

Supplement: Figure S1 — Total feed consumption. Consumption averaged by temperature-set, from either the nesting cage or temperature cage over the six day testing period. LSM and SE are plotted and the diagonal line indicates a significant linear contrast. (TIF) [file pone.0032799.s001.tif]

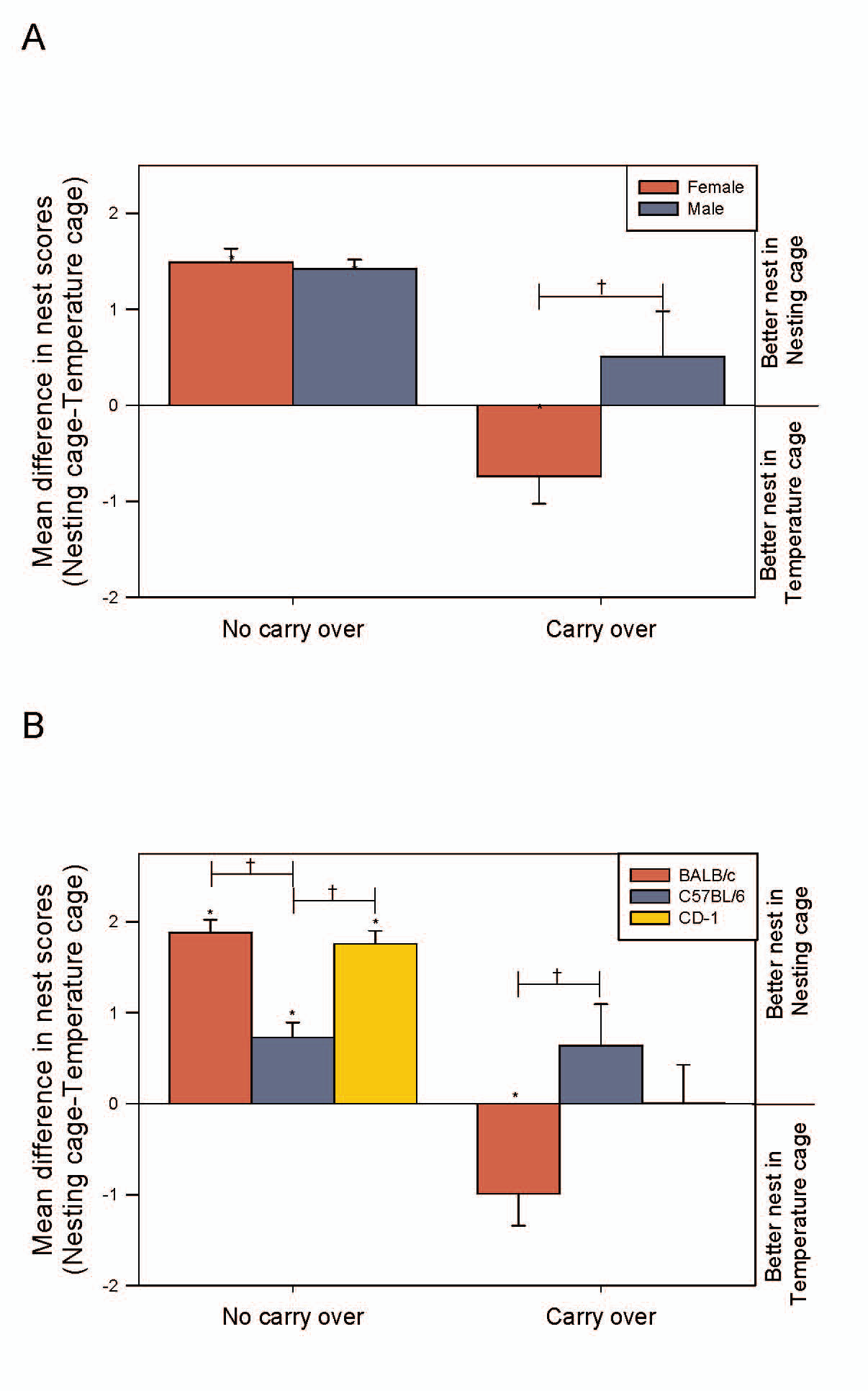

Supplement: Figure S2 — The mean difference in nest score values between the nesting cage and the temperature cage. Nest scores partitioned by occurrences of nesting material carryover by (a) sex; and (b) strain. A negative value indicates a better nest built in the temperature cage and a positive value indicates a better nest in the nesting cage. LSM and SE are plotted and significant Bonferroni corrected planned comparisons are indicated by †. (TIF) [file pone.0032799.s002.tif]

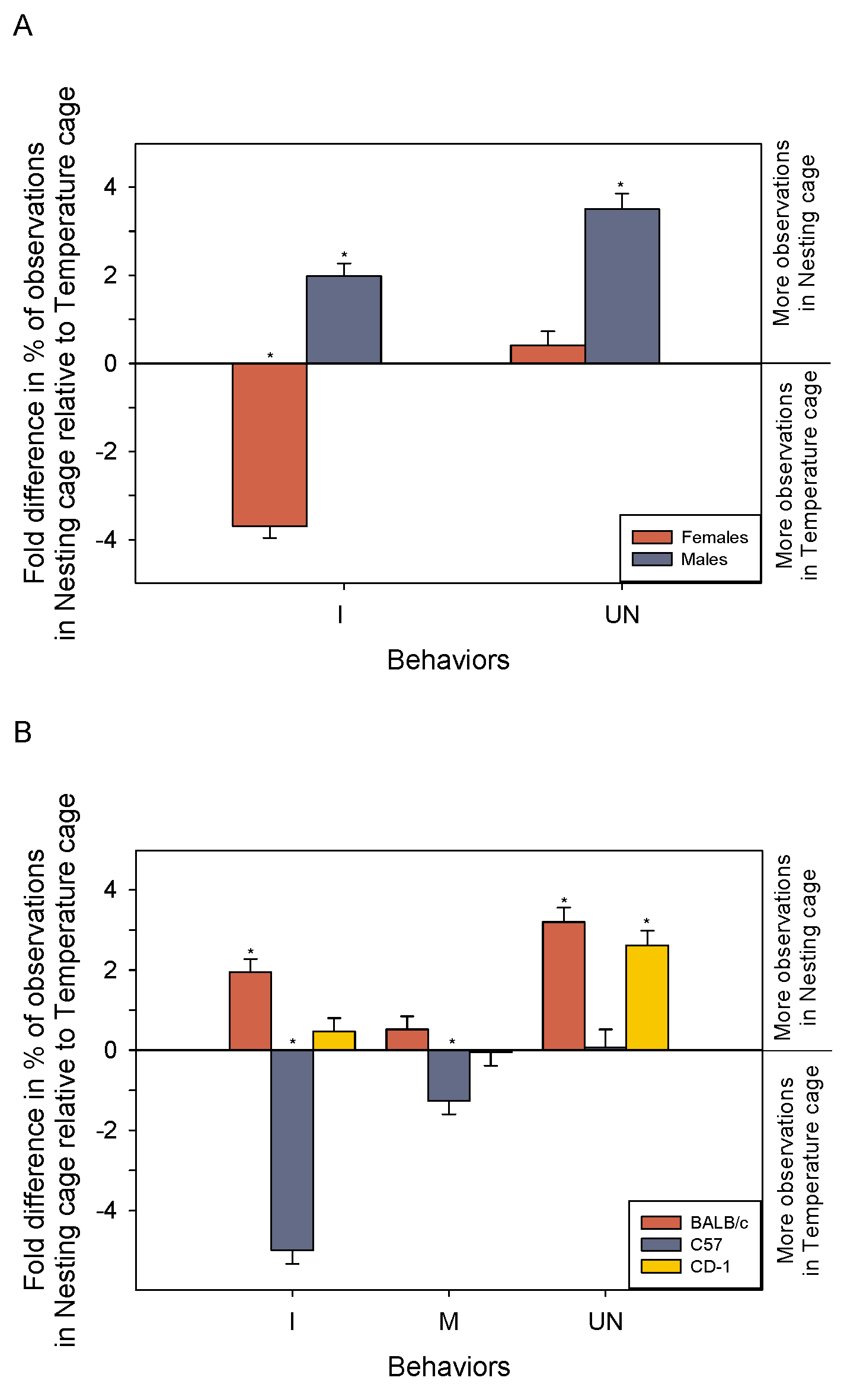

Supplement: Figure S3 — Location preference by behavior. Differences in behavior are plotted by interactions with (a) sex and (b) strain. LSM and SE are plotted and significant t-tests (value different from zero-α corrected for the number of comparisons) are indicated by asterisks. (TIF) [file pone.0032799.s003.tif]
